# Applications of Tert-Butyl-Phenolic Antioxidants in Consumer Products and Their Potential Toxicities in Humans

**DOI:** 10.3390/toxics12120869

**Published:** 2024-11-29

**Authors:** Ngoc M. H. Hoang, Kwangsik Park

**Affiliations:** College of Pharmacy, Dongduk Women’s University, Seoul 02748, Republic of Korea; hoangngoc.hmhn@gmail.com

**Keywords:** tert-butyl phenolic antioxidants, consumer products, exposure, toxicity

## Abstract

Tert-butyl phenolic antioxidants (TBP-AOs) are employed to inhibit oxidation and function as stabilizers and protectants in a broad spectrum of consumer products, such as food packaging, adhesives, lubricants, plastics, and cosmetics. The extensive utilization of TBP-AOs results in human exposure through various pathways. Furthermore, some TBP-AOs have been identified as potential endocrine disruptors and may cause liver and lung damage, as well as allergic reactions. Considering their varied applications and potential toxicity, a detailed evaluation of their safety profiles is imperative. However, existing research is often segmented and tends to focus narrowly on specific compounds. Consequently, this review collates recent data on TBP-AOs regarding their production, exposure, and toxicity, incorporating different databases and prior studies, as well as predictions of toxicity using ADMET. Our review strives to offer a comprehensive overview of the characteristics and health effects of TBP-AOs to guide future research and inform policy decisions.

## 1. Introduction

Antioxidants are agents that can delay or prevent autoxidation by inhibiting the formation of free radicals or by interrupting their propagation. Among these, phenolic antioxidants constitute a significant class of compounds that impede oxidation processes crucial to both commercial and biological systems [1]. These antioxidants are regularly incorporated into consumer products to curb oxidation and enhance shelf life, thereby playing an essential role in preserving the quality and stability of products vulnerable to oxidative degradation.

Within the group of synthetic phenolic antioxidants, numerous compounds resemble tert-butyl phenol. These compounds consist of one or more tert-butyl phenol units, with each comprising at least one tert-butyl group attached to a phenol ring [2]. They have gained widespread use in various consumer goods, including food, cosmetics, and personal care products [2]. Referred to as tert-butyl phenol antioxidants (TBP-AOs), these substances belong to a subclass of synthetic phenolic antioxidants and share common characteristics with other phenolic compounds, such as stabilizing and protecting materials from oxidative degradation. However, TBP-AOs may demonstrate distinctive properties due to their tert-butyl phenol functional groups. Tert-butyl phenol (TBP), with its basic structure of a tert-butyl group linked to a phenol ring, is extensively utilized as an antioxidant, UV stabilizer, and precursor in the manufacture of plastics, rubber, petroleum, paints, coatings, and pharmaceuticals, especially in industrial environments, owing to its high stability [3].

Like most synthetic phenolic antioxidants, TBP-AOs are primarily used in consumer products to protect against environmental oxidative effects. However, the antioxidant properties of TBP-AOs may extend beyond simple preservation due to their capability to combat oxidative stress. The antioxidative properties of TBP-AOs have attracted attention for their potential therapeutic applications in various health conditions. For example, 2,6-di-tert-butylphenol (AO701) derivatives have shown potential neuroprotective effects by reducing glutamate-induced oxidative toxicity in neuronal cells and demonstrating beneficial outcomes in an in vivo rodent model of ischemic stroke, indicating low toxicity and promising therapeutic possibilities [4]. These compounds also inhibit lipid peroxidation induced by mersalyl in rat kidney cortical mitochondria, suggesting a potential protective role against free-radical-mediated nephrotoxicity [5]. Tert-butyl hydroquinone (TBHQ) mitigates oxidative stress and apoptosis in various cell types by enhancing antioxidant enzyme activities and activating the Nrf2 pathway, while also offering protective effects against arsenic toxicity, colorectal cancer metastasis, retinal oxidative damage, cartilage destruction in osteoarthritis, and metabolic issues [6,7,8,9,10,11,12]. 2,4-di-tert-butylphenol (AO33) exhibits a range of protective and therapeutic effects, including antioxidant properties, improved cognitive function in mice, inhibition of viral and bacterial activities, and anti-inflammatory and anticancer effects [13,14,15,16,17,18,19]. These findings indicate that TBP-AOs play a significant role not only in product preservation but also in potential therapeutic strategies for various diseases and conditions associated with oxidative stress.

The widespread use of TBP-AOs in consumer goods underscores their importance, yet it also necessitates a thorough understanding of their potential health and environmental impacts. Although TBP-AOs offer significant benefits in various applications, some of these compounds may induce toxicity in the human body, raising concerns about their safety and the necessity for regulatory scrutiny [2]. Many other TBP-AOs have insufficient safety information or limited research available. This lack of knowledge poses challenges to assessing their potential health risks and environmental impacts. Our review aims to provide a comprehensive examination of the chemical structure, characteristics, uses, current research, and ADMET predictions concerning the health impacts of TBP-AOs. It highlights their critical role in modern industry and daily life while also guiding future research efforts.

## 2. Chemical Structure and Properties

TBP-AOs are characterized by one or more tert-butyl phenol units linked together, with each unit featuring one or more tert-butyl groups, −(C(CH₃)₃), attached to the phenol ring. The phenol group, identified by its hydroxyl (-OH) group bonded to an aromatic ring, is essential in the antioxidant properties of TBP-AOs due to its capacity to donate hydrogen atoms. This donation neutralizes free radicals and reduces oxidative stress [20]. The large tert-butyl group increases the phenol’s stability by providing steric hindrance, protecting it from rapid oxidation [21]. Consequently, these functional groups collaborate to enhance the effectiveness of TBP-AOs in preventing oxidation, making them extremely efficient in various industrial applications where oxidative stability is crucial.

### 2.1. Phenol Group

Phenolic compounds, consisting of one or more phenol groups with different substituents on the aromatic ring, are categorized based on their functional groups. When carboxylic acids are either directly attached or separated by a C=C bond, they are classified as hydroxybenzoic acids or hydroxycinnamic acids, respectively. Compounds with multiple phenol units are termed polyphenols [22]. These compounds exhibit antimicrobial, antioxidant, and anti-inflammatory properties, making them useful in pharmaceuticals for treating cardiovascular disease, diabetes, cancer, and hypertension. They also serve as food preservatives and additives, with additional roles in the cosmetic and packaging industries [22]. In particular, polyphenols, found naturally in fruits and vegetables, are recognized for their therapeutic benefits and technological applications [23]. In the fields of cosmetics and dermatology, phenolic compounds are primarily used for their antioxidant properties, which help prevent premature aging, provide photoprotection, and treat sensitive or sun-damaged skin [24]. In medical and pharmaceutical research, their significant health benefits, including their role as natural chemopreventives for age-related metabolic disorders and their ability to scavenge free radicals, have made phenolic compounds highly esteemed [25].

### 2.2. Tert-Butyl Group

The tert-butyl group, represented by the formula (CH₃)₃C-, is a large alkyl substituent in organic chemistry that significantly influences the structure and properties of the molecules it attaches to [21]. Its considerable size can induce steric hindrance, potentially slowing or inhibiting reactions at adjacent sites. This group is crucial in organic chemistry and is extensively used in both research and industrial settings, particularly in drug synthesis, to alter the chemical and physical properties of various compounds. In commercial products and pharmaceuticals, tert-butyl groups can enhance biological activity by increasing the solubility of compounds in organic solvents, thanks to the hydrophobic nature of its methyl groups, and by stabilizing compounds through the protection of functional groups [26]. In organic synthesis, it frequently serves as a protective group for other functional groups, such as hydroxyl groups, during complex synthesis procedures by providing steric hindrance, protecting sensitive areas in molecules, and minimizing unwanted reactions [27].

### 2.3. Tert-Butyl Phenol

When attached to the aromatic ring, the tert-butyl group profoundly affects the structure and properties of phenol by introducing significant steric hindrance. This hindrance can reduce the reactivity of phenol, particularly in reactions requiring close proximity of reagents to the aromatic ring, such as electrophilic aromatic substitution [28]. The tert-butyl group acts as an electron-donating group through the inductive effect, enhancing the electron density on both the aromatic ring and the hydroxyl group [29,30]. It stabilizes phenolic compounds by slowing oxidation rates, stabilizing phenoxy radicals during antioxidant activity, and increasing effectiveness in preventing oxidation. Additionally, it lowers the acidity of cation radicals, making tert-butylated phenols effective antioxidants [31]. The hydrophobic nature of the tert-butyl group augments the overall hydrophobic character of the molecule, influencing its solubility in various solvents and making it suitable for non-polar environments, such as plastics and oils. Overall, the tert-butyl group markedly enhances the stability, reactivity, and physical properties of phenol derivatives, making tert-butyl phenolic compounds valuable in various industrial applications, particularly as antioxidants and stabilizers.

### 2.4. Classifications

Based on the number of tert-butyl phenol groups present, TBP-AOs are classified into three categories: mono-TBP, di-TBP, and poly-TBP. This classification underscores the diversity of TBP-AOs, allowing for tailored applications based on their antioxidative properties and structural complexity. Table 1 displays the chemical names, structures, and common names of some frequently used TBP-AOs in each category.

### 2.5. Physical Properties

TBP-AOs display a variety of physical properties that vary significantly due to differences in their molecular structures, such as the number of tert-butyl groups and the nature of the attached R-groups. These variations affect important characteristics, including molecular weight, melting point, boiling point, and their physical state at room temperature, which can be either solid or liquid. Table 2 provides a summary of some of these physical properties, as taken from the Chemicalbook database. As shown in Table 2, most TBP-AOs are typically solid and appear white to light yellow, although some can exist as liquids at room temperature. The phenolic group in TBP-AOs imparts weak acidic properties, with pKa values ranging from around 10 to 12. Additionally, most TBP-AOs have a LogP greater than 1, indicating a tendency to dissolve in lipids, which raises concerns about their absorption in the human body. Therefore, caution is advised regarding exposure to TBP-AOs, as their physical properties suggest that they can easily enter biological systems.

## 3. Applications in Consumer Products

TBP-AOs serve as stabilizers and protectants across a broad spectrum of consumer products. They appear in adhesives, sealants, lubricants, greases, plastics, polymers, and rubber used in both industrial and household settings. Moreover, TBP-AOs are found in everyday items, such as detergents, cosmetics, and personal care products, as well as in food coatings. Table 3 presents the applications of some widely used TBP-AOs according to different databases, including the European Chemicals Agency (ECHA), Chemicalbook, and ChemBK.

### 3.1. Adhesives and Sealants

Adhesives and sealants are essential in various industries, notably in non-woven fabrics (such as baby diapers, feminine hygiene products, and medical supplies), tapes, furniture adhesives, and industrial and construction applications. Antioxidants are crucial in preventing the thermal degradation of polymers in adhesives, sealants, and coatings. Hindered phenolic antioxidants, such as Pentaerythritol tetrakis(3-(3,5-di-tert-butyl-4-hydroxyphenyl)propionate) (AO1010) and Octadecyl 3-(3,5-di-tert-butyl-4-hydroxyphenyl)propanoate (AO1076), are preferred for their enhanced thermal stability that boosts processing and durability [32]. Butylated hydroxytoluene (BHT) is commonly used in shoe adhesives and has been linked to allergic contact dermatitis in users [33,34]. By incorporating these antioxidants, manufacturers can enhance the durability and longevity of their products, thus fulfilling the rigorous performance standards of contemporary applications.

### 3.2. Lubricants and Greases

Lubricants and greases are employed to reduce friction, wear, and heat in mechanical components. They are widely used in household items like appliances, vehicles, and tools, as well as in industrial machinery. Lubricants consist of base oils and additives, such as antioxidants and anti-wear agents, to enhance performance. In contrast, greases are semi-solid or solid lubricants [35]. TBP-AOs play a pivotal role in lubricants and greases, enhancing the oxidative resistance of base oils, which improves thermal stability and extends the service life of the lubricant [35,36]. The efficacy of TBP-AOs is attributed to their hindered phenol structure, which protects by donating hydrogen from their phenolic hydroxyl group. This reaction with RO· and ROO· radicals mirrors the mechanism seen in aromatic amine molecules, effectively inhibiting oxidative degradation in lubricating oils [36].

### 3.3. Plastics, Polymers, and Rubber

Polymers, plastics, and rubbers are vital materials in everyday life, offering versatility and functionality across a wide range of applications. These materials are found in common items, such as packaging, containers, household appliances, toys, and vehicle components. TBP-AOs significantly enhance the durability and performance of these items. For example, Tris(2,4-di-tert-butylphenyl) phosphite (AO168), a widely used antioxidant in plastics, has been found to contaminate various laboratory reagents, potentially biasing ecotoxicological and toxicological studies. Additionally, oxidized AO168 has been detected in reverse osmosis and deionized water containers [37]. AO701 and 4,4′-bis(2,6-di-tert-butylphenol) are commonly employed as antioxidants in the manufacturing of rubber to prevent degradation from oxygen and heat exposure, thus enhancing product durability and performance [38,39]. Other TBP-AOs, like 1,3,5-trimethyl-2,4,6-tris(3,5-di-tert-butyl-4-hydroxybenzyl) benzene (AO1330), AO1010, and AO1076, are used to stabilize crosslinked and non-crosslinked polyethylene materials [40,41]. Like other phenolic antioxidants, TBP-AOs protect these materials through various mechanisms. They serve as thermal stabilizers, safeguarding against degradation due to high temperatures, and they protect against UV damage by neutralizing free radicals [42,43]. Additionally, they mitigate oxidative degradation by neutralizing oxygen radicals, helping to maintain the polymers’ flexibility and elasticity, which is essential for applications involving repeated flexing, thus extending product lifespan [44]. Moreover, they delay aging processes, such as embrittlement and cracking, particularly in outdoor environments, while preserving mechanical strength and elongation properties to ensure consistent performance under demanding conditions [45].

### 3.4. Cosmetics and Personal Care Products

The increasing interest in both natural and synthetic antioxidants for use in cosmetics and personal care products is fueled by their ability to shield the skin from oxidative stress and prevent age-related damage. TBP-AOs, known for their antioxidant properties, have become essential components in skincare and beauty formulations. Compounds like 2-tert-butyl-4-methoxyphenol (BHA), BHT, and TBHQ are integrated into a diverse range of cosmetics and personal care products, such as foam stabilizers [46], hair dyes [47], lipsticks, eye shadows, blushers, and skin creams [48,49]. In addition to their antioxidative capabilities, certain TBP-AOs also inhibit tyrosinase, an enzyme critical for melanin production, and boost glutathione reductase activity, making them effective for use in skin-lightening and anti-pigmentation products [50,51,52,53,54].

### 3.5. Food and Coatings

Foods are prone to various oxidative reactions initiated by specific enzymes or molecular oxygen, which generate reactive free radicals. These reactions can lead to undesirable flavors and odors, reduced nutritional value, and diminished shelf life of products. Antioxidants play a crucial role as additives that impede these oxidative processes, thus prolonging the shelf life of foods without altering their intrinsic properties. They are employed in food packaging and coatings that are in direct contact with food items. Additives in these materials may stay within the polymer, degrade due to environmental influences, or migrate into the food, potentially reducing the effectiveness of antioxidants in polymers and posing safety risks to humans via food consumption. Therefore, rigorous standards for the stability and safety of these food additives are essential. Natural phenolic compounds are extensively used in food packaging, as well as in edible films and coatings [55]. TBP-AOs, including AO1076, AO1010 [56,57], and AO168 [58,59], are frequently utilized as antioxidant additives in food packaging polymers to counteract polymer degradation caused by oxygen, light, and heat due to their durability and safety.

### 3.6. Other Applications

TBP-AOs are utilized in various sectors beyond food packaging, where they perform critical functions as antioxidants and preservatives. For example, AO1076 is a commonly used phenolic antioxidant in several polymer-based medical devices, such as catheters, to protect against microbial contamination [60,61]. Santowhite (STW) serves in cage implant systems to prevent surface cracking and flaking [62]. In pharmaceutical containers, the use of BHT, AO168, and AO1010 may introduce risks to patients or affect the quality of the product [63]. Moreover, compounds like AO1010, Benzenepropanamide, N,N’-1,6-hexanediylbis(3,5-bis(1,1-dimethylethyl)-4-hydroxy- (AO1098), and 1,3,5-tris[(3,5-ditert-butyl-4-hydroxyphenyl)methyl]-1,3,5-triazinane-2,4,6-trione (AO3114) aid in reducing the formation of malodor molecules in fabrics during the use of laundry detergents [56].

## 4. Human Exposure

TBP-AOs are found in various daily use items. Consequently, human exposure to these compounds can occur through multiple pathways, each with distinct risks based on the nature of contact, including dermal contact, inhalation, oral intake, and environmental exposure. The extent of human exposure to TBP-AOs varies considerably due to factors like geographic location, age, personal characteristics, consumption patterns, and the degree to which these chemicals penetrate products, the body, and the environment. Despite the importance of these variations, existing data on TBP-AO exposure remain insufficient. Some exposure routes for TBP-AOs include the following.

### 4.1. Dermal Contact

TBP-AOs are found in personal care products, cosmetics, and plastics. Direct application or dermal contact during use can lead to absorption of TBP-AOs. The extent to which these substances are absorbed, retained in human skin, and penetrate the circulation has not been thoroughly investigated. However, research using a consumer-like dermal exposure model showed that ten different polymer additives, including 4,4′-Thiobis(6-tert-butyl-m-cresol) (AO300), 2,2′-Methylenebis(4-methyl-6-tert-butylphenol) (AO2246), 2,2′-Thiobis(6-tert-butyl-p-cresol) (AO1081), and Diethyl 3,5-di-tert-butyl-4-hydroxybenzylphosphonate (AO1222), can penetrate and distribute within the intact porcine and human skin barriers depending on their lipophilicity [64]. A study on the dermal absorption of AO300 found that approximately 20% of the applied dose was absorbed in mice, with rats showing significantly lower absorption rates, under 2% [65]. The dermal absorption rate of BHT in animal skin models varies, ranging from 0.07% in pig skin to 11.1% in fuzzy rat skin [66]. Higher absorption rates may contribute to increased systemic exposure and potential toxicity, making it crucial to understand these rates when evaluating the safety and potential health risks of these compounds in consumer products. In humans, daily dermal intake of TBP-AOs varies with different daily contact with items, such as personal care products and paperboard food packages [67,68]. Additionally, TBP-AOs may induce allergic contact dermatitis upon exposure to items containing these compounds, such as cosmetics [69], gloves [70], wound bandages [71], medical devices [72,73], and other contact materials.

### 4.2. Inhalation Exposure

TBP-AOs are widely used in industrial settings, particularly in the production of paints, adhesives, plastics, and rubber. The use of TBP-AOs in these applications can lead to inhalation exposure for workers and individuals near manufacturing environments. During the production and application processes, TBP-AOs may be released into the air as vapors or particulates, posing potential respiratory risks. Workers who handle these materials may be particularly susceptible to exposure, potentially experiencing a range of health effects, including respiratory irritation and other systemic impacts. A 49-year-old non-atopic male factory worker developed asthma while cleaning mixing drums containing TBHQ, a common additive in food and cosmetics that can cause allergic contact dermatitis, suggesting that TBHQ may be the cause of his asthma [74]. TBP-AOs, such as AO1076, AO168, and their oxidation products, were detected in the atmosphere where plastic waste was incinerated, contributing to air pollution and increasing the risk of inhalation exposure to TBP-AOs [75].

### 4.3. Oral Exposure

For many years, researchers have been studying the oral exposure and potential toxic effects of additives that leach from consumer products into food or body fluids. TBP-AOs, commonly found in materials that contact food, can transfer from packaging or storage containers into the food itself. Gao et al. discovered high migration levels of TBP-AOs, including BHT, BHA, AO2246, Irganox 1035 (AO1035), AO1010, and AO1330, with BHT and AO2246 notably exceeding specific migration levels in certain food simulants [76]. These chemicals exhibit substantial migration levels, raising concerns about potential TBP-AO exposure through food intake. This poses possible health risks and suggests that glass containers might be a safer alternative to plastic for storing oil-based foods. Monitoring and determining the specific migration levels of these additives is crucial for ensuring food quality. Additionally, handling materials containing TBP-AOs and then consuming food without washing hands could also lead to ingestion.

The estimated daily intake (EDI) of specific TBP-AOs from food sources has been examined in numerous studies, revealing considerable variability influenced by various factors, such as the region, the subjects, and the types of foods containing these compounds. This variability underscores the significance of context in evaluating exposure levels. As summarized in Table 4, various studies report differing EDI values for certain TBP-AOs, reflecting regional exposure differences. Further research is essential to provide more comprehensive data on the EDI of other TBP-AOs across diverse populations and food sources.

### 4.4. Gestational Exposure

Gestational exposure to environmental chemicals, including TBP-AOs, poses significant health risks to both pregnant women and their developing fetuses. TBP-AOs can cross the placental barrier and potentially impact fetal development. A recent study investigated prenatal exposure to 46 plasticizers and antioxidants in 109 pairs of maternal and cord serum samples to assess the ability of these chemicals to cross the placenta [89]. The results indicated that TBP-AOs, such as AO33 and BHT, along with their primary transformation products, demonstrated relatively high transplacental transfer efficiencies [89]. The transformation products of BHT exhibit greater potential for maternal transfer compared to BHT itself, whereas AO2246 and AO33 were found in significant levels in cord plasma and placenta, rather than in maternal blood [90]. These findings emphasize that TBP-AOs, including AO33, can permeate the placental barrier, suggesting potential adverse effects on fetal development. This underscores the need for further research and regulatory measures to mitigate prenatal exposure to harmful environmental chemicals.

### 4.5. Environmental Exposure

TBP-AOs persist in the environment and can contaminate the air, soil, and water through industrial waste or degradation of consumer products. Research on antioxidants in household dust from various locations has revealed the widespread presence of TBP-AOs, such as Triethylene glycol bis(3-tert-butyl-4-hydroxy-5-methylphenyl)propionate (AO245), 2,6-di-tert-butyl-4-(dimethylaminomethyl)phenol (AO703), AO33, AO701, BHA, and BHT, as well as their derivatives, across different regions [91,92]. Individuals residing in contaminated areas might be exposed to TBP-AOs through dust, drinking water, or the food chain, leading to an increased risk of health issues. A study exploring the potential endocrine-disrupting effects of the migrating compound AO33 suggested that migration from plastic pipes could lead to prolonged exposure, with significant variations in migration levels depending on the type of plastic pipe and the manufacturer [93]. Additionally, 4-tert-butylphenol (PTBP), used as a hardener in epoxy resins, can leach from steel coatings upon contact with water and induce acute estrogenic effects [94].

Certain TBP-AOs, although initially considered safe, can undergo environmental transformations resulting in highly toxic byproducts. For instance, AO701, deemed non-toxic by European Union standards, forms a degradation product through photodegradation under natural sunlight, 2,5-di-tert-butylphenol, that exhibits significant toxicity to marine bacteria [95]. Consequently, the environmental impact of these compounds may be more severe than initially assessed, necessitating a comprehensive evaluation of their behavior and transformation in natural waters.

### 4.6. Occupational Exposure

In addition to daily exposure, occupational exposure to TBP-AOs and their derivatives significantly increases the risk of toxicity. Workers in industries that manufacture or use these compounds may encounter higher concentrations, thereby elevating the risk of acute and chronic health effects. Table 5 presents the production volume and the total number of workers exposed to specific TBP-AOs in 2019, as reported to the U.S. Environmental Protection Agency (EPA) by various companies, along with the annual volume noted by the European Chemicals Agency (ECHA). These data underscore the potential extent of exposure and associated health risks, emphasizing the need for rigorous monitoring and regulation of occupational exposure to TBP-AOs to safeguard workers’ health. Implementing safety measures is critical to minimize the risks these chemicals pose in the workplace.

In summary, human exposure to TBP-AOs can occur through dermal contact, inhalation, oral ingestion, and environmental exposure, with each posing specific health risks. To mitigate these risks, it is critical to regulate TBP-AO usage, develop safer alternatives, and ensure safe working environments.

## 5. Human Health Effects

The toxicity of TBP-AOs is a significant concern due to their widespread use in various applications and the risk of migration from materials to food, pharmaceuticals, and other consumer products. Studies have indicated that some TBP-AOs may cause adverse effects, including skin toxicity, liver toxicity, lung toxicity, and endocrine disruption. In the skin, TBP-AOs can lead to atopic dermatitis and depigmentation in the skin. In the liver, these compounds may cause hepatotoxic effects, including changes in liver enzyme activity, disruption of metabolic functions, and induction of cell death. In the lung, certain TBP-AOs are linked to asthma, pneumotoxicity, tumor development, and lung damage. Furthermore, in the endocrine system, TBP-AOs might disrupt normal hormone functions and potentially lead to reproductive toxicity.

While research on the toxic effects of TBP-AOs in humans is still sparse, some in vitro and in vivo studies have been undertaken to assess their toxicological profiles and potential mechanisms of action. However, the complete range of these toxicities and their mechanisms is still not fully understood, with only a few TBP-AOs having been thoroughly investigated regarding their toxicity and safety profiles. Below is a summary of current insights into the impact of TBP-AOs on human health.

### 5.1. Skin Toxicity

Atopic dermatitis and depigmentation are significant skin toxicities associated with TBP-AOs. Numerous studies have demonstrated that TBP-AOs can cause allergic contact dermatitis when individuals come into contact with consumer products containing these compounds, such as shoes [96], cosmetics [69], and medical devices [70,71,72,73]. While the precise mechanism remains unclear, several patients have developed allergic contact dermatitis following exposure to latex medical devices that contain AO300 [70,72], BHA [70], and 2-tert-Butyl-6-(3-tert-butyl-2-hydroxy-5-methylbenzyl)-4-methylphenyl acrylate (AO3052) [73]. Furthermore, PTBP can exacerbate atopic-dermatitis-like skin lesions in mice by enhancing T-helper 2-type immune responses and increasing inflammatory cytokines, which suggests its potential to aggravate allergic skin conditions [97]. The presence of TBP-AOs in various products raises concerns about their potential to trigger or exacerbate atopic dermatitis in susceptible individuals, underscoring the importance of carefully evaluating these compounds in consumer products.

Depigmentation is another skin toxicity linked to TBP-AOs, particularly PTBP. This compound has been found to induce oxidative stress and apoptosis in melanocytes, leading to depigmentation disorders, such as vitiligo and leukoderma [98,99]. The oxidative damage and endoplasmic reticulum stress in melanocytes highlight the severe effects PTBP can have on skin health, contributing to significant issues like cutaneous depigmentation and exacerbation of atopic dermatitis. Additionally, PTBP derivatives, including TBHQ, 4-hydroxyanisole, and 4-tert-butyl catechol, which are commonly used in consumer applications, have caused depigmentation in animal models [50,100]. Notably, 4-tert-butyl catechol has been shown to stimulate the formation of pheomelanosomes in skin melanocytes by activating glutathione-metabolizing enzymes and inhibiting the oxidation of eumelanin intermediates [101]. Furthermore, cases of cutaneous depigmentation have also been reported in occupational exposures to AO33 in the rubber industry [102].

### 5.2. Liver Toxicity

TBP-AOs can induce various hepatotoxic effects, including modulation of liver enzymes, disruption of hepatic metabolism, apoptosis, and necrosis. For example, PTBP has been shown to impair liver development and function in zebrafish, resulting in liver damage, increased lipid accumulation, altered activities of metabolic enzymes, and changes in the expression of key metabolic and inflammatory genes, ultimately leading to tissue dysfunction [103]. In addition, PTBP can induce apoptosis, necroptosis [104], and ferroptosis [105], highlighting its potential to cause significant liver damage.

In contrast, BHT presents a complex profile regarding liver toxicity. BHT is renowned for its capacity to prevent chemically induced tumors and reduce the acute toxic effects of various chemicals through multiple mechanisms [106,107,108,109,110]. Owing to its antioxidant capabilities, BHT has been used as a reference compound in studies assessing the efficacy of antioxidants in defending against oxidative stress and associated damage [108]. Additionally, BHT offers protective effects against liver damage induced by specific carcinogens, such as diethylnitrosamine [107] and aflatoxin B1 [109,110]. However, prolonged exposure to BHT can result in hepatotoxic effects, characterized by elevated liver enzymes and histopathological alterations [111]. It also influences the expression of genes associated with both phase I and phase II metabolism in the liver, highlighting its potential effects on hepatic function and the significance of gene expression profiling in comprehending the toxicity of food additives [112]. Moreover, BHT exacerbates liver injury and oxidative stress when administered in conjunction with LPS [113].

Some TBP-AOs also exhibit hepatotoxic effects. For example, BHA and AO2246 disrupt hepatic lipid homeostasis and fatty acid levels [114,115]. 2,2′-Methylenebis(4-ethyl-6-tert-butylphenol) (AO425) adversely affects mitochondrial function and enzyme activity in the liver [116]. Meanwhile, 2,4,6-Tri-tert-butylphenol (AO246) causes liver injury characterized by focal necrosis and microcytic anemia, especially in female rats, and it does not induce neoplastic responses [117]. The complex and often multifaceted nature of liver toxicity associated with various TBP-AOs underscores the need for a comprehensive understanding of their mechanisms of action and effects.

### 5.3. Lung Toxicity

Exposure to TBP-AOs and their derivatives in daily life, particularly through inhalation, raises concerns about their toxicity to the respiratory system. Pneumotoxicity has been widely observed with BHT, especially in relation to tumor development and lung damage.

BHT has exhibited dose-dependent pneumotoxicity in CD-1 mice, resulting in respiratory distress, lung congestion, alveolar collapse, dilated alveolar ducts, and epithelial cell degeneration or necrosis, while no similar effects were observed in BHA-treated mice or other tested species [118]. However, BHA intensifies BHT-induced lung toxicity in mice by increasing hydrogen peroxide formation and promoting the peroxidase-dependent conversion of BHT into a toxic metabolite [119]. Furthermore, BHT serves as a non-genotoxic tumor promoter, notably enhancing tumor development when combined with a carcinogen, although it does not independently induce tumors [120]. It can foster lung tumor growth through the induction of chronic inflammation [121], an increase in reactive oxygen species, the deactivation of key antioxidant enzymes [122], and the activation of functional TLR4 [123]. This resulting inflammation is instrumental in tumor promotion and increases lung tumor multiplicity when paired with carcinogen treatment.

Conversely, TBHQ demonstrates dose- and time-dependent cytotoxic and genotoxic effects on lung cancer cells and endothelial cells, leading to apoptosis and DNA fragmentation [124] or causing cytotoxicity through the electrophilic reactions of its metabolites [125]. A recent study has associated TBHQ with respiratory issues, such as asthma, highlighted by a case where a factory worker developed asthma symptoms following exposure [74]. While numerous studies have examined the respiratory system impacts of certain TBP-AOs, the safety of other compounds in this group remains unverified. Considering the frequent use of TBP-AOs in industrial applications, workers and individuals near these areas might be at risk of inhalation exposure. Further research is imperative to assess the risk of respiratory toxicity of other TBP-AOs and their potential health implications.

### 5.4. Endocrine Disruption

Endocrine disruption refers to the interference of chemicals with the body’s hormonal systems. Several TBP-AOs, particularly PTBP, BHT, and BHA, have been identified as endocrine disruptors. TBP-AOs can act as either agonists or antagonists to key hormones, such as testosterone, androgens, estrogen, and progesterone, thereby impacting normal endocrine function. These compounds may mimic hormonal action, activate receptors, or block natural hormones from exerting their effects, particularly when influencing aromatase activity and altering estrogen levels. However, the effects of some TBP-AOs on hormone levels are inconsistent among studies.

For instance, PTBP binds to estrogen receptors, promoting cell proliferation and enhancing the expression of estrogen-regulated proteins, such as the progesterone receptor and pS273, albeit with minimal estrogen antagonism [126]. PTBP reduces estradiol and testosterone secretion in isolated ovarian follicles from rats without affecting aromatase activity, highlighting its potential impact on steroidogenesis in follicles [127]. However, other studies have shown that PTBP markedly increases testosterone and progesterone levels by up to sevenfold in fetal rat testes [128] and inhibits aromatase in JEG-3 cells [129].

Additionally, BHT and BHA are well-documented for their potential to disrupt endocrine systems, significantly affecting hormonal balance and reproductive health. In T47D-Kbluc and MCF-7 breast cancer cells, BHT and BHA exhibited both estrogenic and anti-estrogenic activities [130]. Another study on pregnant mice demonstrated that BHT increases estrogen and progesterone levels at 200 mg/kg/day [131]. Conversely, another study found that BHA, BHT, and AO2246 did not bind directly to ERα or exhibit estrogenic effects; instead, they notably enhanced E2 secretion by disrupting steroidogenic processes in the human adenocarcinoma cell line, H295R [132]. Moreover, BHA and BHT displayed anti-androgenic properties by inhibiting DHT-induced luciferase activity in a concentration-dependent manner in the androgen receptor of MDA-kb2 human breast cancer cells [133].

Finally, the modulation of the endocrine system by TBP-AOs can lead to reproductive toxicity. In males, BHT disrupts calcium homeostasis and induces endoplasmic reticulum stress in Leydig cells, resulting in testicular toxicity and adversely affecting male reproductive health [134]. In females, studies involving immature and ovariectomized rodents have indicated that TBP-AOs, such as BHA, AO300, and STW, can induce uterotrophic activity following repeated treatment and dietary exposure [135,136]. Additionally, exposure to BHT during pregnancy significantly decreased maternal body weight, the number of implantation sites, and uterine weight, while increasing serum levels of estrogen and progesterone, indicative of potential reproductive toxicity [131].

The endocrine disruption effects of TBP-AOs, as summarized in Table 6, have been recognized in various studies. Although some TBP-AOs are identified as potential endocrine disruptors, research into their endocrine-disrupting effects and underlying mechanisms remains sparse. Existing studies offer some insight into how TBP-AOs may influence key hormones, such as testosterone, androgens, estrogen, and progesterone; however, the specific pathways and mechanisms are still largely undefined. This lack of a comprehensive understanding underscores the urgent need for further research to elucidate the effects of TBP-AOs on hormonal systems and their role in endocrine disruption. Detailed investigations are crucial to determine the mechanisms of action of TBP-AOs and their potential health risks, particularly concerning reproductive and metabolic disorders. The impacts of TBP-AOs on the endocrine system show considerable variation across studies, influenced by various factors such as cell types and experimental models, highlighting the need for standardized methods to evaluate their endocrine-disrupting potential.

## 6. Prediction of Toxicities Using ADMET

Many TBP-AOs are currently in use and are anticipated to be employed in various applications in the future. Nevertheless, research on their toxicity remains inadequate, raising concerns about potential health hazards. To address this issue, we utilize ADMETlab 2.0 “https://admetmesh.scbdd.com/ (accessed on 27 September 2024)”. as a crucial tool for evaluating the toxicological characteristics of these compounds. The method for using ADMETlab 2.0 has been described in a prior study [137]. In this review, predicted probability values are represented by three symbols indicating the likelihood of a compound being active: (_) for low probability (probability score: 0–0.3), (+) for medium probability (probability score: 0.3–0.7), and (+++) for high probability (probability score: 0.7–1). This classification allows for a rapid assessment of the compounds’ toxicological profiles. Employing ADMET models enables us to understand their potential harmful effects, subsequently guiding future research to ensure safer applications and inform regulatory decisions. The predicted toxicity of TBP-AOs from ADMET analysis is presented in Table 7. According to these predictions, most TBP-AOs are likely to induce skin and eye irritation and respiratory toxicity and impact endocrine regulation and cellular stress responses. However, there is a notable absence of comprehensive studies on these toxicities of TBP-AOs. This research gap underscores the urgent need for further investigation to fully understand their potential health implications. Specifically, ADMET predictions indicate that many TBP-AOs might affect estrogen regulation, as confirmed by several studies. Moreover, the antioxidative properties of TBP-AOs could modulate key pathways involved in oxidative stress, inflammation, and cellular protection. Understanding these interactions is vital for determining the overall impact of TBP-AOs on health and disease, as well as their potential risks and benefits. As predicted, TBP-AOs may influence peroxisome proliferator-activated receptors (PPARs), which are crucial in lipid metabolism and inflammatory responses [138,139], as well as cellular responses to stress and protection mechanisms, such as the antioxidant response element (ARE), the heat shock factor response element (HSE), and mitochondrial membrane potential (MMP). Given these potential impacts, investigating how TBP-AOs affect these pathways and their implications for human health is essential. Consequently, additional research is needed to explore the effects of TBP-AOs on these processes.

## 7. Future Perspective

Aside from TBP-AOs, many other antioxidants are being explored for their similar protective properties while presenting lower health risks. These alternatives aim to maintain product stability and safety without compromising consumer health. Natural antioxidants, such as vitamin C, vitamin E, phenolic acids, phenolic diterpenes, and flavonoids extracted from fruits and plants, provide excellent protection against oxidation [140]. These substances are generally recognized as safe and have demonstrated effectiveness in various applications, including food and cosmetics. Additionally, novel antioxidants, like peptides, proteins, and enzymes, show promise for use in food preservation, cosmetics, and therapeutics [141,142]. However, these compounds have some limitations, such as low antioxidant activity, instability under temperature, moisture, and oxygen, and high costs associated with extraction and purification processes. Therefore, synthetic antioxidants, including TBP-AOs, are still widely used in consumer products. Research is ongoing to develop modified versions of these compounds that retain their effectiveness while reducing potential health risks.

As TBP-AOs are widely used in consumer products and the demand for effective stabilizers grows, TBP-AOs are likely to see expanded use in food packaging, cosmetics, and industrial formulations owing to their antioxidant, physical, and biological properties. This increased usage will necessitate greater regulatory scrutiny and comprehensive risk assessments. However, the safety profiles of these compounds are not well-understood, raising concerns about potential health impacts. There is an urgent need for comprehensive research on the toxicological effects of TBP-AOs and standardized testing methods to elucidate their biological effects.

Continued research into their toxicity is essential for shaping industry policies and practices, ultimately ensuring enhanced protection for public health and the environment. Comprehensive information gathering is required, including extensive literature reviews and meta-analyses on their safety and efficacy, development of standardized testing protocols to assess their toxicological profiles, and establishment of clear guidelines for their safe use in consumer products. By implementing these strategies, we can deepen our understanding of the implications of TBP-AOs and ensure their use is consistent with public health and environmental safety priorities.

## Figures and Tables

**Table 1:** Classification and common TBP-AOs.

Group	No.	Chemicals	Structure	Common Name	Abbreviation	CAS. No
Mono-TBP	1	4-Tert-butylphenol	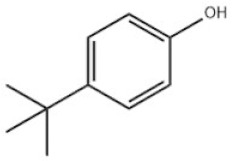	PTBP	PTBP	98-54-4
	2	2-Tert-Butyl-4,6-dimethylphenol	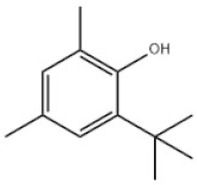	Antioxidant AO30	AO30	1879-09-0
	3	2,6-Di-tert-butylphenol	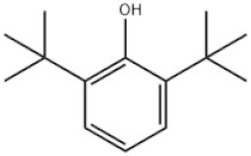	Ethanox 701	AO701	128-39-2
	4	Butylated Hydroxytoluene	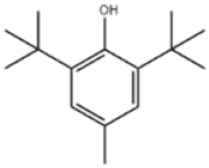	BHT	BHT	128-37-0
	5	2,6-Di-tert-Butyl-4-(dimethylaminomethyl)phenol	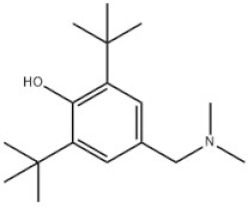	Antioxidant 703	AO703	88-27-7
	6	Diethyl 3,5-di-tert-butyl-4-hydroxybenzylphosphonate	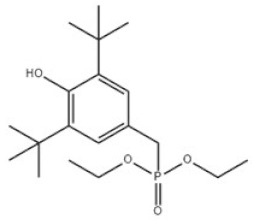	Antioxidant 1222	AO1222	976-56-7
	7	Octadecyl 3-(3,5-ditert-butyl-4-hydroxyphenyl)propanoate	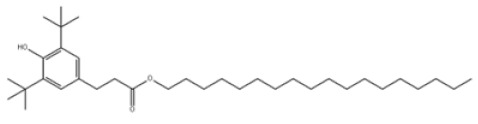	Antioxidant 1076	AO1076	2082-79-3
	8	4-((4,6-Bis(octylthio)-1,3,5-triazin-2-yl)amino)-2,6-di-tert-butylphenol	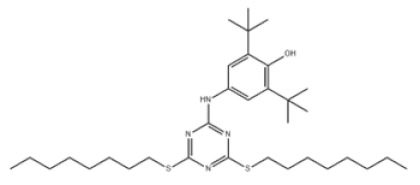	Antioxidant 565	AO565	991-84-4
	9	Calcium bis(ethyl 3,5-di-tert-butyl-4-hydroxybenzylphosphonate)	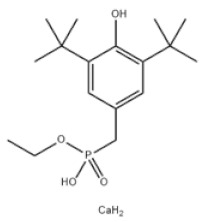	Antioxidant 1425	AO1425	65140-91-2
	10	2,4,6-Tri-tert-butylphenol	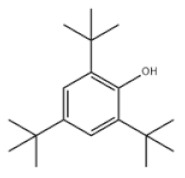	Antioxidant 246	AO246	732-26-3
	11	2-Tert-Butyl-4-methoxyphenol	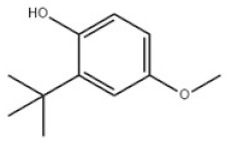	3-BHA	BHA	121-00-6
	12	2,4-Di-tert-butylphenol	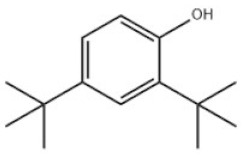	Antioxidant 33	AO33	96-76-4
	13	Tert-Butylhydroquinone	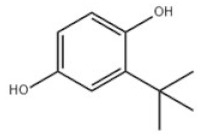	TBHQ	TBHQ	1948-33-0
	14	3,5-Di-tert-butyl-4-hydroxybenzyl alcohol	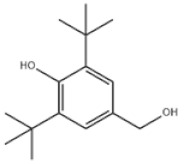	Antioxidant 754	AO754	88-26-6
	15	Octyl-3,5-di-tert-butyl-4-hydroxy-hydrocinnamate	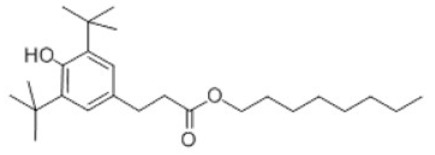	Antioxidant 1135	AO1135	125643-61-0
	16	11-Methyldodecyl 3-(3,5-di-tert-butyl-4-hydroxyphenyl)propanoate	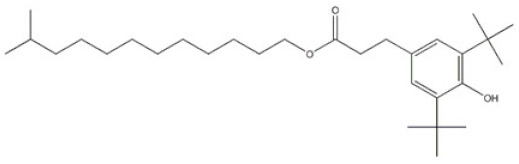	Antioxidant 1077	AO1077	847488-62-4
	17	4-Sec-Butyl-2,6-di-tert-butylphenol	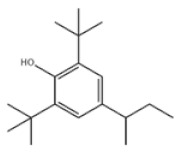	ISONOX 132	AO132	17540-75-9
	18	Methyl 3-(3,5-di-tert-butyl-4-hydroxyphenyl)propionate	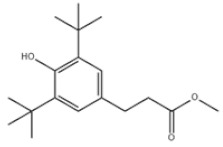	Ralox 35	AO35	6386-38-5
	19	6-Tert-Butyl-m-cresol	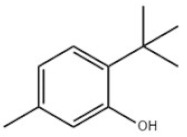		TBMC	88-60-8
Di-TBP	20	2,2′-Methylenebis(4-methyl-6-tert-butylphenol)	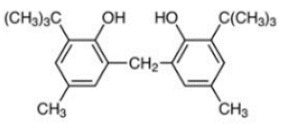	Antioxidant 2246	AO2246	119-47-1
	21	2,2′-Thiobis(6-tert-butyl-p-cresol)	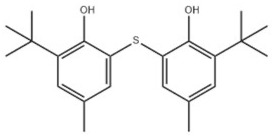	Antioxidant 1081	AO1081	90-66-4
	22	2,2′-Methylenebis(4-ethyl-6-tert-butylphenol)	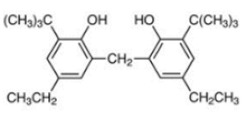	Antioxidant 425	AO425	88-24-4
	23	Santowhite	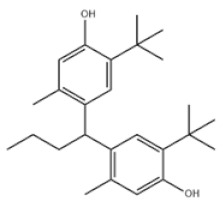	Santowhite	STW	85-60-9
	24	2-Tert-Butyl-6-(3-tert-butyl-2-hydroxy-5-methylbenzyl)-4-methylphenyl acrylate	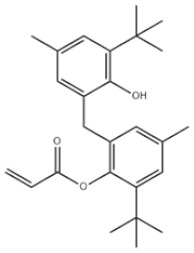	Antioxidant 3052	AO3052	61167-58-6
	25	4,4′-Methylenebis(2,6-Di-tert-butylphenol)	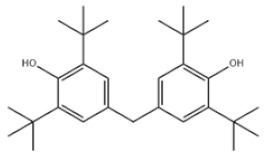	Antioxidant 702	AO702	118-82-1
	26	1,2-Bis(3,5-di-tert-butyl-4-hydroxyhydrocinnamoyl)hydrazine	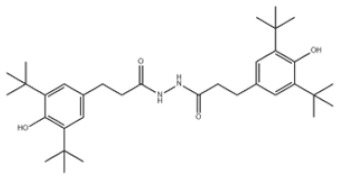	Antioxidant 1024	AO1024	32687-78-8
	27	Triethylene glycol bis(3-tert-butyl-4-hydroxy-5-methylphenyl)propionate	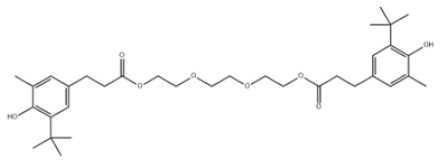	Antioxidant 245	AO245	36443-68-2
	28	N,N’-Propane-1,3-diylbis[3-(3,5-DI-tert-butyl-4-hydroxyphenyl)propionamide]	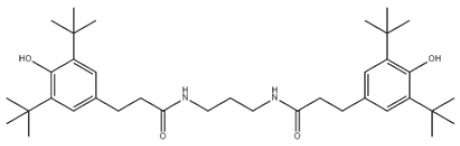	Antioxidant 1019	AO1019	69851-61-2
	29	3,9-Bis(2,4-di-tert-butylphenoxy)-2,4,8,10-tetraoxa-3,9-diphosphaspiro[5.5]undecane	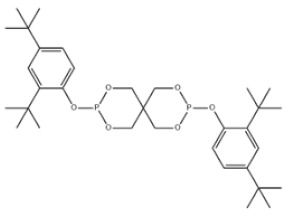	Ultranox 626	AO626	26741-53-7
	30	Bis(2,6-di-tert-butyl-4-methylphenyl)pentaerythritol diphosphite	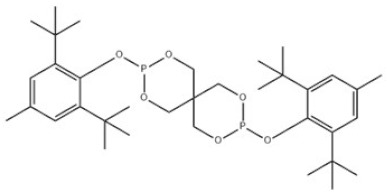	Antioxidant PEP-36	AO36	80693-00-1
	31	Hexamethylene bis[3-(3,5-di-tert-butyl-4-hydroxyphenyl)propionate]	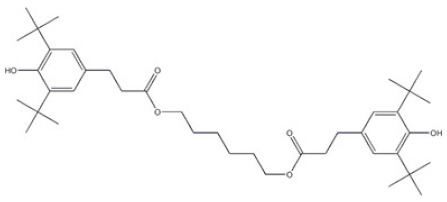	Irganox 259	AO259	35074-77-2
	32	Irganox 1035	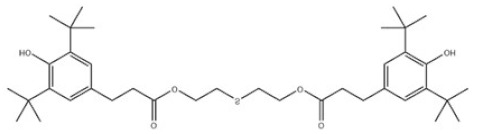	Antioxidant 1035	AO1035	41484-35-9
	33	Sumilizer AG 80	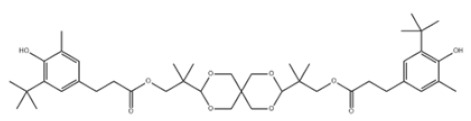	Antioxidant AO80	AO80	90498-90-1
	34	2,2′-Ethylidenebis(4,6-di-tert-butylphenol)	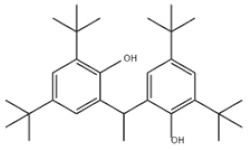	Antioxidant 1290	AO1290	35958-30-6
	35	Benzenepropanamide, N,N’-1,6-hexanediylbis(3,5-bis(1,1-dimethylethyl)-4-hydroxy-	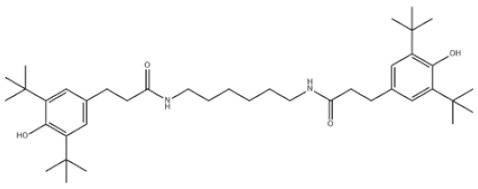	Antioxidant 1098	AO1098	23128-74-7
	36	Naugard XL-1	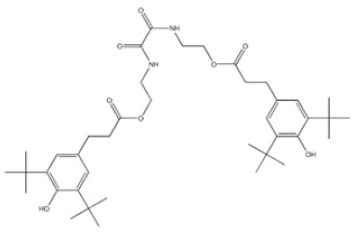	Antioxidant MD-697	AO697	70331-94-1
	37	4,4′-Thiobis(6-tert-butyl-m-cresol)	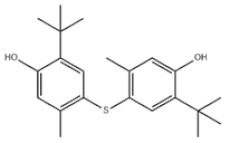	Antioxidant 300	AO300	96-69-5
Poly-TBP	38	1,1,3-Tris(2-methyl-4-hydroxy-5-tert-butylphenyl)butane	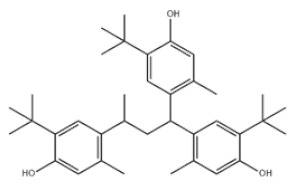	Antioxidant CA	AOCA	1843-03-4
	39	Tris(2,4-di-tert-butylphenyl) phosphite	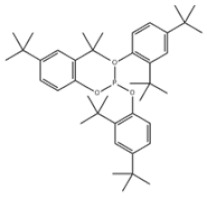	Antioxidant 168	AO168	31570-04-4
	40	1,3,5-Triazine-2,4,6(1H,3H,5H)-trione, 1,3,5-tris[[4-(1,1-dimethylethyl)-3-hydroxy-2,6-dimethylphenyl]methyl]-	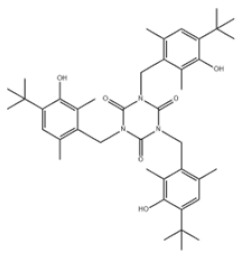	Antioxidant 1790	AO1790	40601-76-1
	41	1,3,5-Trimethyl-2,4,6-tris(3,5-di-tert-butyl-4-hydroxybenzyl)benzene	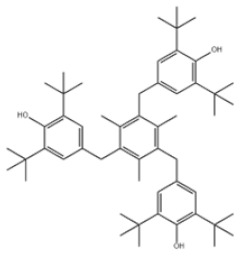	Antioxidant 1330	AO1330	1709-70-2
	42	1,3,5-Tris[(3,5-ditert-butyl-4-hydroxyphenyl)methyl]-1,3,5-triazinane-2,4,6-trione	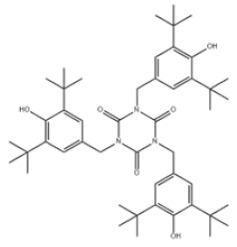	Antioxidant 3114	AO3114	27676-62-6
	43	Pentaerythritol tetrakis(3-(3,5-di-tert-butyl-4-hydroxyphenyl)propionate)	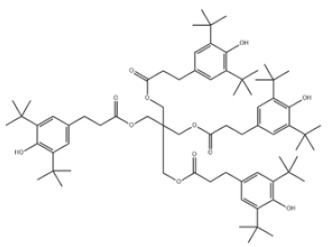	Antioxidant 1010	AO1010	6683-19-8

**Table 2:** Physical properties of some TBP-AOs.

No.	Compounds	MW (g/mol)	Melting Point	Boiling Point	Form	Color	LogP	pKa
1	PTBP	150.22	96–101 °C	236–238 °C	Flakes or pastilles	White to light beige	3 at 23 °C	10.23 at 25 °C
2	AO30	178.27	22 °C	249 °C	Powder to lump to clear liquid	White or colorless to light yellow	3.64 at 35 °C	12.00 ± 0.23
3	AO701	206.32	34–37 °C	253 °C	Crystalline solid	White to light yellow	4.5 at 24 °C	12.16 ± 0.40
4	BHT	220.35	69–73 °C	265 °C	Crystals	White	5.2	14 (uncertain)
5	AO703	263.42	93–94 °C	172 °C at 30 mm Hg	Powder to crystal	White to yellow to orange	4.24	11.17 ± 0.70
6	AO1222	356.44	122 °C	417.0 ± 33.0 °C	Powder to crystal	White to almost white	2.9 at 23 °C	12.04 ± 0.40
7	AO1076	530.86	50–52 °C	568.1 ± 45.0 °C	-	White to off-white	13.930	12.33 ± 0.40
8	AO565	588.95	92–95 °C	670.7 ± 65.0 °C	Solid	White to off-white	13.2 at 25 °C	12.27 ± 0.70
9	AO1425	370.48	-	-	-	-	−0.1 at 23 °C	-
10	AO246	262.43	125–130 °C	277 °C	Buffered aqueous glycerol solution	Off-white to pale yellow	7.1 at 35 °C	12.61 ± 0.40
11	BHA	180.24	58–64 °C	269 °C	Powder/waxy solid	White to off-white	1–2.82 at 25–27 °C	11.83 ± 0.18
12	AO33	206.32	53–56 °C	265 °C	Crystalline solid	White to yellow	4.8 at 23 °C	11.56 ± 0.18
13	TBHQ	166.22	127–129 °C	295 °C	Crystalline powder	White to light tan; may contain black specks	1.521 at 25 °C	10.80 ± 0.18
14	AO754	236.35	139–141 °C	214 °C at 40 mm Hg	Solid: particulate/powder	White to yellow to orange	3.16 at 20 °C	12.01 ± 0.40
15	AO1135	390.6	-	343–370.65 °C at 101 325 Pa	Oil	Colorless	7.18–9.2 at 0–30 °C	-
16	AO1077	460.73	-	220–245 °C at 101.3 kPa	Viscous	-	3.56 at 25 °C	-
17	AO132	262.43	25 °C	141–142 °C at 10 mm Hg	Liquid	White or colorless to yellow	7.2 at 20 °C	11.85 ± 0.70
18	AO35	292.41	60–67 °C	125–130 °C (Press: 0.1 Torr)	Powder to crystal	White to almost white	4.866	12.17 ± 0.40
19	TBMC	164.24	20 °C	117–118 °C at 12 mm Hg	-	Colorless to red to green	3.97	11.45 ± 0.10
20	AO2246	340.5	123–127 °C	187 °C	Solid	White to off-white	6.25 at 20 °C	11.32 ± 0.48
21	AO1081	358.54	84–85 °C	431.1 ± 45.0 °C	-	-	6.5 at 25 °C	10.30 ± 0.50
22	AO425	368.55	119–122 °C	458.86 °C	Solid	White to off-white	8.95 at 20 °C	11.37 ± 0.48
23	STW	382.58	211 °C	469.7 °C	Powder to crystal	White to almost white	6.4 at 20 °C	10.44 ± 0.20
24	AO3052	394.55	128–133 °C	491 °C	Solid	White to off-white	-	11.66 ± 0.48
25	AO702	424.66	155–159 °C	289 °C at 40 mm Hg	solid	White	8.9 at 25 °C	12.03 ± 0.40
26	AO1024	552.79	60–67 °C	652.6 ± 55.0 °C	Solid	White to off-white	4.8 at 23 °C	11.10 ± 0.50
27	AO245	586.77	79–81 °C	602.88 °C	Solid	White to off-white	4.7 at 23 °C	11.44 ± 0.25
28	AO1019	594.87	-	711.0 ± 60.0 °C	-	-	8.13	12.08 ± 0.40
29	AO626	604.69	160–178 °C	555.8 ± 50.0 °C	Solid	White to off-white	10.9 at 25 °C	-
30	AO36	632.75	235–240 °C	577.0 ± 50.0 °C	-	-	6 at 25 °C	-
31	AO259	638.92	102–105 °C	648.1 ± 55.0 °C	Solid	Off-white	-	12.03 ± 0.40
32	AO1035	642.94	78 °C	659.4 ± 55.0 °C	Solid	White to off-white	-	12.02 ± 0.40
33	AO80	740.98	125 °C	755.7 ± 55.0 °C	Solid	White to off-white	-	11.44 ± 0.25
34	AO1290	438.68	162–164 °C	464.8 ± 40.0 °C	-	-	9.840	11.28 ± 0.50
35	AO1098	636.95	156–161 °C	740.1 ± 60.0 °C	Solid	White to off-white	9.6 at 25 °C	12.08 ± 0.40
36	AO697	696.91	178 °C	-	Solid	White to off-white	8.1 at 35 °C	11.48 ± 0.46
37	AO300	358.54	160–165 °C	460.94 °C	Powder	White to off-white	5.24 at 25 °C	10.76 ± 0.36
38	AOCA	544.81	183–190 °C	578.54 °C	-	-	12.7 at 25 °C	10.38 ± 0.20
39	AO168	646.94	181–184 °C	594.2 ± 50.0 °C	Powder	White	18 at 25 °C	-
40	AO1790	699.92	163–165 °C	793.8 ± 60.0 °C	Solid	White to off-white	15.281 at 20 °C	11.36 ± 0.28
41	AO1330	775.2	248–250 °C	739.54 °C	Solid	White to off-white	17.17	11.91 ± 0.40
42	AO3114	784.08	218–220 °C	757.9 ± 60.0 °C	-	White to off-white	15.18	11.45 ± 0.40
43	AO1010	1177.66	115–118 °C	779.1 °C	Solid	White to off-white	18.832	11.71 ± 0.40

All data were taken from Chemicalbook (https://www.chemicalbook.com/) on 24 November 2024. MW: molecular weight; pKa: negative logarithm of the acid dissociation constant; LogP: logarithm of the partition coefficient.

**Table 3:** Consumer uses of some common TBP-AOs.

No.	Compounds	Consumer Products and Materials Using TBP-AOs		
		ECHA	Chemicalbook	ChemBK
1	PTBP	Sealants, adhesives, and coatings.	Sealants, adhesives, coating products, polymers, and other materials.	
2	AO30	Fuels, hydraulic fluids, metal working fluids, lubricants, and greases.	Jet and rocket fuels.	Fuel, acrylic acid polymerization, and pharmaceuticals.
3	AO701	Fuels, lubricants, and greases.	Fuels, lubricants, plastics, rubber, and polymers.	Fuel, rubber and plastic, UV absorber, and pesticide and dye applications.
4	BHT	Cleaning products, plant protection, lubricants, greases, adhesives, sealants, polishes, waxes, coating, and fertilizers.	Oils and fat-containing foods.	Rubber, plastic, gasoline, oil, and food.
5	AO703		Rubber, synthetic resin, gasoline, and oil.	
6	AO1222			Polyester, polycondensation, dimethyl terephthalate, polyamide, and UV absorber applications.
7	AO1076	Coatings, lubricants, greases, adhesives, sealants, polishes, waxes, air care, and cleaning products.	Plastics, synthetic fibers, elastomers, adhesives, waxes, oils, fats, polyolefin, and other polymers.	Resin, rubber, petroleum, polyolefin, and polyvinyl chloride.
8	AO565	Adhesives and sealants.	Resin, elastomers, adhesives, polystyrene, polyamide, and polyolefin.	Resins, elastomers, rubber, adhesives, and ABS plastic.
9	AO1425	Rosin, resin, and polymers (*).		
10	AO246		Electromagnetic bushing.	Rubber.
11	BHA	Cosmetics and personal care products.	Cosmetics, topical medications, foods, fuels, rubber, plastics, paints, and glues.	Organic raw materials, biochemical reagents, etc.
12	AO33	Fuels.	Polyolefins, UV stabilizers, pharmaceuticals, and fragrances.	Used as a chemical intermediate, light stabilizer, UV absorber, and plasticizer.
13	TBHQ		Cosmetics, edible fats and vegetable oils, potato chips, and dry cereal.	Cosmetics, edible oils and fats, lard, frying foods, rubber, resin, plastics, pharmaceuticals, etc.
14	AO754		Gasoline and other hydrocarbons.	
15	AO1135		Polymers, food packaging, and textile staining.	Rubber, elastomer, metal deactivators, polyether, polyurethane, oil.
16	AO1077			Plastics, synthetic fiber, waxes, greases, elastomers, and rubber.
17	AO132		Polyols, PVC, adhesives, and functional fluids.	
18	AO35	Cosmetics, biocides, fragrances, polishes, waxes, cleaning, air care, and personal care products.	Polyethylene.	
19	TBMC		Lubricating oil and others.	Organic synthesis intermediate.
20	AO2246	Fuels, adhesives, sealants, lubricants, greases, and hydraulic and metal working fluids.	Distilled biodiesel.	Rubber, latex, other materials, and petroleum products.
21	AO1081			Rubber and polymer materials.
22	AO425			Rubber and synthetic resin.
23	STW	Coatings, adhesives, and sealants.	Polyolefin and rubber.	
24	AO3052		Adhesive, plastic, and elastomer materials.	Cosmetics, food processing, plastics, rubber, paint, drugs.
25	AO702	Coatings, lubricants and greases, and washing and cleaning products.		Polymers and resin.
26	AO1024		Packaging polymers, resins, adhesives, and food contact.	Polyolefin materials, wires, cables, and insulating materials.
27	AO245		Adhesives and polymers for food contact.	Rubber, latex, resins, and polymers.
28	AO1019	Wires and cables and metal-contact materials (*).		
29	AO626	Coatings, adhesives, sealants, inks, and toners.	Food contact, rubber, elastomers, coatings, adhesives, polymers, and plastics.	Polymer materials.
30	AO36		Plastics.	
31	AO259	Lubricants and greases.		
32	AO1035		Wire, cable, polymers, resins, and adhesives.	Coatings, plastics, and rubber.
33	AO80		Polymers, plastics, rubber, adhesives, sealants, etc.	
34	AO1290			Act as antioxidant.
35	AO1098		Plastics, adhesives, elastomers, polymers, and fibers.	Polymers, resin, and rubber.
36	AO697		Cables, polymers, resin, and other materials.	
37	AO300		Rubber, plastics, and food contact polymers.	Polyethylene packaging film, rubber, resin, etc.
38	AOCA	Adhesives and sealants.	Polymers, resins, and light-colored rubber products.	
39	AO168	Coatings, adhesives and sealants, inks, and toners.	Polymers, resin, plastics, binding agent, rubber, and petroleum.	Polymers, fiber, resin, and other plastics.
40	AO1790		Polystyrene and rubber-modified polystyrene in food contact.	Nylon, pipes, agricultural films, household appliances, polymers, resin, and plastics.
41	AO1330	Adhesives, sealants, lubricants, and greases.	Polymers, elastomers, fibers, adhesives, waxes, oils, and fats	Polymers, plastics, resin, and rubber.
42	AO3114	Adhesives and sealants.	Nonfatty food packaging and propylene copolymers.	Polymers.
43	AO1010	Coatings, adhesives, sealants, lubricants, greases, polishes, waxes, and cleaning and air care products.	Plastics, fibers, elastomers, adhesives, waxes, oils, and fats.	Polymers, resin, and plastic products.

(*) Intended use, but not mentioned in databases. ECHA: “https://www.epa.gov/ (accessed on 23 September 2024)”. Chemicalbook: “https://www.chemicalbook.com/ (accessed on 23 September 2024)”. ChemBK: https://www.chembk.com/ (accessed on 23 September 2024)”.

**Table 4:** Summary of estimated daily intake (EDI) of certain TBP-AOs from foods.

Compounds	Mean EDI (mg/kg bw/day)	Subjects	Country	Year	Ref.
BHA	0.14–0.17	16,014 households	Brazil	1997–1999, 2003	[77]
	5.49–12.12 (mg/person/day)	13,000 individuals of all ages	Canada	1973	[78]
	0–0.04	11,525 individuals of all ages	Korea	1998	[79]
	0.001–0.017	3003 individuals of all ages	France	1998–1999	[80]
	0–0.72	706 children (aged 1–36 months)	France	2005	[81]
	5.1 × 10^−4^–0.3	134 samples	Korea	2005	[82]
	0.15	230 children (aged 9–18 years)	Lebanon	2002–2003	[83]
BHA and/or BHT	0.075	5898 individuals of all ages	The Netherlands	1987/1988	[84]
BHT	0.09–0.11	17,014 households	Brazil	1997–1999, 2003	[77]
	0.13–0.39	13,000 individuals of all ages	Canada	1973	[78]
	1.56 × 10^−5^–0.04	11,525 individuals of all ages	Korea	1998	[79]
	0–0.013	3003 individuals of all ages	France	1998–1999	[80]
	0–0.267	441–4079 individuals of all ages	France, Italy, the UK, Ireland	1992–2007	[85]
	0.018–0.025	230 children (age 9–18 years)	Lebanon	2002–2003	[83]
	7.5 × 10^−4^–0.29	131 samples	Korea	2005	[82]
	0.003–0.087	32 different dietary surveys on peoples of all ages	Europe (22 countries)	2010	[86]
	1.4 × 10^−03^	Snack and cookie samples	China	2019	[67]
	2.51 × 10^−5^–4.71 × 10^−5^	1780 individuals (aged 6 months–17 years)	Spain	2012–2014	[87]
	6.61 × 10^−3^	952 individuals of all ages	China	2021	[88]
TBHQ	0.11–0.14	18 014 households	Brazil	1997–1999, 2003	[77]
	1.2 × 10^−6^–0.04	11,525 individuals of all ages	Korea	1998	[79]
	2.5 × 10^−4^–0.28	104 samples	Korea	2005	[82]
AO1076	1.07 × 10^−3^	Snack and cookie samples	China	2019	[67]
AO2246	7.81 × 10^−5^	Snack and cookie samples	China	2019	[67]
AO245	1.40 × 10^−5^	Snack and cookie samples	China	2019	[67]

**Table 5:** Production summary of TBP-AOs.

No.	Chemicals	EPA		ECHA
		Total Exposed Workers *	2019 National Aggregated Production (lbs.)	Annual Manufactured and Import Volume (tons)
1	PTBP	235–515	20,000,000–100,000,000	≥10,000
2	AO30	25–60	1,000,000–20,000,000	100–1000
3	AO701	75–220	100,000,000–1,000,000,000	1000–10,000
4	BHT	905–2265	1,000,000–10,000,000	10,000–100,000
5	AO703	10–35	<1,000,000	100–1000
6	AO1222	-	-	10–100
7	AO1076	970–2700	20,000,000–100,000,000	≥10,000
8	AO565	25–80	100,000–<500,000	100–1000
9	AO1425	10–45	<1,000,000	100–1000
10	AO246	75–160	20,000,000–100,000,000	100–1000
11	BHA	-	-	≥10
12	AO33	125–260	20,000,000–100,000,000	≥1000
13	TBHQ	-	<1,000,000	100–1000
14	AO754	-	-	1–10
15	AO1135	620–1390	10,000,000–50,000,000	-
16	AO1077	-	-	10–100
17	AO1320	25–49	1,000,000–20,000,000	10–100
18	AO35	3000–5996	20,000,000–100,000,000	1–100
19	TBMC	25–49	1,000,000–20,000,000	-
20	AO2246	700–1750	1,000,000–10,000,000	1000–10,000
21	AO1081	-	-	10–100
22	AO425	<10	<1,000,000	1–10
23	STW	-	100,000–500,000	100–1000
24	AO3052	-	-	10–100
25	AO702	50–110	1,000,000–20,000,000	100–1000
26	AO1024	50–130	1,000,000–10,000,000	100–1000
27	AO245	200–1040	1,000,000–10,000,000	1000–10,000
28	AO1019	-	-	10–100
29	AO626	50–120	1,000,000–20,000,000	1000–10,000
30	AO36	<10	138713	10
31	AO259	500–1020	<1,000,000	100–1000
32	AO1035	500–1020	1,000,000–20,000,000	100–1000
33	AO80	<10	<1,000,000	-
34	AO1290	60–125	<1,000,000	-
35	AO1098	50–110	<1,000,000	1000–10,000
36	AO697	50–100	<1,000,000	100–1000
37	AO300	50–110	100,000–500,000	100–1000
38	AOCA	1050–10130	100,000–500,000	100–1000
39	AO168	1985–4995	10,000,000–50,000,000	10,000–100,000
40	AO1790	75–160	1,000,000–20,000,000	100–1000
41	AO1330	100–240	1,000,000–20,000,000	1000–10,000
42	AO3114	75–180	1,000,000–10,000,000	1000–10,000
43	AO1010	2070–5490	50,000,000–100,000,000	≥10,000

*: The categorization of workers reasonably likely to be exposed to chemicals was reported as a range with 9 options: 0–10, 10–25, 25–50, 50–100, 100–500, 500–1000, 1000–10,000, more than 10,000, and NKRA (Other and Not Known or Reasonably Ascertainable). The total number of workers exposed to a specific chemical is calculated by summing the upper and lower limits of the worker ranges reported by all companies producing that chemical. EPA: Environmental Protection Agency. ECHA: European Chemicals Agency.

**Table 6:** Summary of endocrine disruption by TBP-AOs.

Compounds	Model	Treatment		Testosterone (LOEL)		Androgen		Aromatase		Progesterone (LOEL)		Estrogen (LOEL)		Ref.
		Dose	Period	AGO	ANT	AGO	ANT (IC50)	AGO	ANT (IC50)	AGO	ANT	AGO	ANT	
PTBP	Isolated fetuses’ testes	10–500 mg/L	24 h	100 mg/L	-	-	-	-	-	100 mg/L	-	-	-	[128]
	Isolated immature rat ovarian follicles	0.01–1 μM	3 days	0.01 μM	0.1 μM	-	-	-	-	-	-	-	0.1 μM	[127]
			5 days	-	0.1 μM	-	-	N	-	-	-	-	0.01 μM	
	MCF-7 cells	10 nM–10 μM	24 h	-	-	-	-	-	-	-	-	-	10 μM	[126]
	JEG-3 cells	5–500 μM	24 h	-	-	-	-	-	283 μM	-	-	-	-	[129]
BHT	T47D-Kbluc cells	0.3–200 μM	24 h	-	-	-	-	-	-	-	-	N	IC25 = 15,734 μM	[130]
	MCF-7 cells	0.3–200 μM	24 h	-	-	-	-	-	-	-	-	10 μM	N	
	MDA-kb2 cells	0.3–300 μM	24 h	-	-	N	43.2 μM	-	-	-	-	-	-	[133]
	Pregnant CD1 mice	200,400 mg/kg/d	6 days	-	-	-	-	-	-	200 mg/kg/d	-	200 mg/kg/d	-	[131]
	H295R cells	1–100 μM	48 h	N	-	N	-	-	-	-	N	100 μM	-	[132]
BHA	T47D-Kbluc cells	0.3–100 μM	24 h	-	-	-	-	-	-	-	-	100 μM	IC50 = 100.22 μM	[130]
	MCF-7 cells	0.3–200 μM	24 h	-	-	-	-	-	-	-	-	EC25 = 5.53 μM, EC40 = 8.96 μM	IC50 = 116.83 μM	
	H295R cells	1–100 μM	48 h	N	-	N	-	-	-	-	N	1 μM	-	[132]
	Zebrafish gonads	1–5 μM	21 days	1 μM	-	-	-	-	-	-	-	1 μM	-	
	MDA-kb2 cells	0.3–300 μM	24 h	-	-	N	172.5 μM	-	-	-	-	-	-	[133]
TBHQ	H295R cells	0.01–1 μM	48 h	N	-	N	-	-	-	-	N	N	-	[132]
AO2246	H295R cells	0.01–1 μM	48 h	N	-	N	-	-	-	-	N	0.1 μM	-	
AO701	JEG-3 cells	50–200 μM	24 h	-	-	-	-	-	N	-	-	-	-	[129]
AO246	JEG-3 cells	3–320 μM	24 h	-	-	-	-	-	58 μM	-	-	-	-	

N: No significant difference. LOEL: Lowest Observed Effect Level. IC50: Inhibitory Concentration 50%. EC50: Effective Concentration 50%.

**Table 7:** Potential for toxicity induction predicted using ADMET.

No.	Compounds	Skin Sensitivity	Eye Corrosion/ Eye Irritation		Respiratory	Endocrine Disruption					PPARs	ARE	HSE	MMP
						Androgen Receptor		Aromatase	Estrogen Receptor					
			EC	EI		AR	AR-LBD		ER	ER-LBD				
1	PTBP	+++	+++	+++	+				+++	+++			+	+++
2	AO30	+++	+++	+++	+						+			+
3	AO701	+++	+++	+++	+				+		+++			+++
4	BHT	+++	+++	+++	+						+++			+++
5	AO703	+++	+++		+++							+		+++
6	AO1222	+	+	+++				+++			+++	+		+++
7	AO1076	+++		+++	+				+		+	+		+
8	AO565	+++		+++	+++			+++	+		+++	+++	+++	+++
9	AO1425			+++				+++	+		+++	+++		+++
10	AO246	+	+++	+++	+				+	+	+++	+		+++
11	BHA	+++	+++	+++	+++					+		+		+++
12	AO33	+++	+++	+++	+				+	+++		+	+	+++
13	TBHQ	+++	+++	+++	+				+++	+++		+++	+++	+++
14	AO754	+++		+++							+++	+	+	+++
15	AO1135	+++		+++	+++			+	+		+++	+		+++
16	AO1077	+++		+++	+++				+		+	+		+++
17	AO132	+		+++	+				+		+++			+++
18	AO35	+		+							+++			+++
19	TBMC	+	+++	+++	+								+	+++
20	AO2246	+++		+++	+				+		+++	+	+++	+++
21	AO1081	+++		+++	+++			+	+		+++	+	+++	+++
22	AO425	+++		+++				+	+++		+++	+++	+++	+++
23	STW	+++		+++	+++			+++	+++	+++	+++	+++	+++	+++
24	AO3052	+++		+++	+++			+	+	+	+++	+++	+++	+++
25	AO702	+++		+++	+			+	+++	+	+++	+	+	+++
26	AO1024	+++							+		+++	+++	+++	+++
27	AO245	+++		+				+	+		+++	+++		+++
28	AO1019	+++							+		+++	+++	+	+++
29	AO626	+		+++	+++			+				+		+++
30	AO36			+++	+++			+						+
31	AO259	+++		+++				+	+	+	+++	+	+	+++
32	AO1035	+++		+++				+	+	+	+++	+	+	+++
33	AO80							+	+	+	+	+		+++
34	AO1290	+++		+++	+			+	+++	+++	+++	+	+	+++
35	AO1098	+++							+		+++	+++	+	+++
36	AO697	+++						+++	+		+++	+++	+	+++
37	AO300	+		+++	+++			+++	+++	+++		+++	+++	+++
38	AOCA	+++		+++	+			+++	+++	+++	+++	+++	+++	+++
39	AO168	+++		+++				+	+	+				+++
40	AO1790			+++				+++		+		+++	+++	+++
41	AO1330	+++		+++				+	+++	+	+	+	+++	+++
42	AO3114			+++				+++			+++	+++	+++	+++
43	AO1010			+++					+	+++	+	+	+++	+++

+++: high probability of being active (probability score: 0.7–1). +: medium probability of being active (probability score: 0.3–0.7). (blank): low probability of being active (probability score: 0–0.3). EC/EI: eye corrosion/irritation; AR/ER: androgen/estrogen receptor; AR/ER-LBD: Androgen/Estrogen Receptor Ligand Binding Domain; PPARs: peroxisome proliferator-activated receptors; ARE: antioxidant response element; HSE: heat shock factor response element; MMP: mitochondrial membrane potential.

## Data Availability

Data sharing is not applicable.
